# ATHENA: A knowledge-based hybrid backpropagation-grammatical evolution neural network algorithm for discovering epistasis among quantitative trait Loci

**DOI:** 10.1186/1756-0381-3-5

**Published:** 2010-09-27

**Authors:** Stephen D Turner, Scott M Dudek, Marylyn D Ritchie

**Affiliations:** 1 Center for Human Genetics Research, Departments of Molecular Physiology & Biophysics and Biomedical Informatics, Vanderbilt University, Nashville, TN, USA

## Abstract

**Background:**

Growing interest and burgeoning technology for discovering genetic mechanisms that influence disease processes have ushered in a flood of genetic association studies over the last decade, yet little heritability in highly studied complex traits has been explained by genetic variation. Non-additive gene-gene interactions, which are not often explored, are thought to be one source of this "missing" heritability.

**Methods:**

Stochastic methods employing evolutionary algorithms have demonstrated promise in being able to detect and model gene-gene and gene-environment interactions that influence human traits. Here we demonstrate modifications to a neural network algorithm in ATHENA (the Analysis Tool for Heritable and Environmental Network Associations) resulting in clear performance improvements for discovering gene-gene interactions that influence human traits. We employed an alternative tree-based crossover, backpropagation for locally fitting neural network weights, and incorporation of domain knowledge obtainable from publicly accessible biological databases for initializing the search for gene-gene interactions. We tested these modifications *in silico *using simulated datasets.

**Results:**

We show that the alternative tree-based crossover modification resulted in a modest increase in the sensitivity of the ATHENA algorithm for discovering gene-gene interactions. The performance increase was highly statistically significant when backpropagation was used to locally fit NN weights. We also demonstrate that using domain knowledge to initialize the search for gene-gene interactions results in a large performance increase, especially when the search space is larger than the search coverage.

**Conclusions:**

We show that a hybrid optimization procedure, alternative crossover strategies, and incorporation of domain knowledge from publicly available biological databases can result in marked increases in sensitivity and performance of the ATHENA algorithm for detecting and modelling gene-gene interactions that influence a complex human trait.

## Background

### Genome-Wide Association Studies, Complex Disease, and Epistasis

The genome-wide association study (GWAS) is a widely used technique in human genetics research to investigate DNA variations associated with common human diseases. The last several decades have ushered in technological advances that have allowed investigators to progress from coarse genomic coverage with linkage maps and candidate gene association studies, to very high resolution association studies using single nucleotide polymorphisms (SNPs). The initial completion and ongoing development of the International HapMap Project [1,2] catalogs common human genetic variation at millions of polymorphic sites in several diverse human populations, facilitating more powerful and strategic association study designs. Several contemporary genotyping technologies enable rapid, highly accurate genotyping of up to millions of common SNPs at low cost per genotype [3].

We have yet to fully explore the abundance of data generated by the studies made possible by these advances in genotyping technology in part because maturation of our analytical strategies for data of this scale have not kept pace. The most commonly used analytical procedures for analyzing GWAS data are very simple tests of association looking at one SNP at a time. This approach has been somewhat successful in identifying genetic variants associated with complex traits, including age-related macular degeneration [4], type II diabetes [5], hypertension [6], and blood cholesterol levels [7,8], among others [9]. However, these single SNPs collectively explain little of the genetic contribution to the trait variance that is expected based on family and twin studies [10]. For instance, HDL-cholesterol level is highly under genetic control - up to 73% of variation in HDL can be explained by genetic factors [11] - yet even the most highly powered genetic studies examining a single SNP at a time found that collectively only ~5% of this variance could be accounted for by single-SNP analysis [7]. Many agree that a portion of this "missing heritability" likely lies in gene-gene and gene-environment interactions [10,12,13]. Indeed, it is well accepted that common traits are complex, and are likely influenced by an elaborate interplay of multiple genetic and environmental factors [14-16]. This is attributed in part to the nature of biomolecular interactions that are essential for regulation of gene expression and complex metabolic networks, and are likely to play a role in influencing human traits [17]. Moreover, several recent perspectives have emphasized that most true single locus genetic associations to complex traits carry a vanishingly small effect size [18,19], and experimental data from model organisms illustrates that gene-gene interaction is pervasive and often carries surprisingly large effects [20,21].

Compelling evidence suggests that gene-gene interaction exists and influences complex traits in both humans and model organisms, yet there is no consensus on how to best examine existing GWAS data for gene-gene interactions that may be influencing the trait of interest. One approach is to evaluate multi-SNP combinations for potential interactive effects based on biological criteria [22]. This may include, for instance, testing interactions between genes that share a similar structure or function, or genes in the same pathway or biological process, such as a receptor and its ligand. Using this strategy would bias the statistical analysis in favor of models with a well-established biological foundation in the literature, and novel biology would remain undiscovered. Furthermore, the entire analysis depends upon the quality of the biological information used. Another approach is to select SNPs based on the strength and statistical significance of their independent main effects, evaluating interactions only between SNPs that meet a certain effect size or significance threshold [23]. This strategy makes the simplifying but false assumption that statistical interactions affecting the outcome can only occur between variants that independently have a detectable effect on the phenotype.

Another strategy to search for influential gene-gene or gene-environment interaction is to exhaustively evaluate the relationship between the outcome of interest and every possible combination of genetic and environmental exposures. While one may wish to fit standard regression models to every possible 2-, 3-, or n-way combination of SNPs, this approach becomes problematic for several reasons. First, when interactions among multiple genetic and/or environmental components are considered, there are many combinations that are present in only a few samples or perhaps none at all. This is known as the curse of dimensionality [24], and results in unstable estimates of population parameters from large-sample based methods. Furthermore, while interpreting the statistical significance of models fit using traditional methods is fairly straightforward, correction must be made for multiple testing. Tests of interactions are large in number and are not independent, making multiple testing corrections difficult. Finally, regardless of the statistical issues associated with exhaustive interaction testing, the computational burden is enormous - there are 1.25 × 10^11 ^two-SNP models among 500,000 SNPs - the number typically represented on contemporary GWAS platforms. Memory issues aside, it would take many years on a desktop computer to run this analysis. Parallel processing drastically reduces this computational burden but does not eliminate it. As the number of three-way interactions in such a dataset is over 2 × 10^16^, searching exhaustively for higher order interactions would be infeasible even on multiprocessor computing clusters. This limitation is the motivation for developing techniques that still utilize the full dimensionality of the data without exhaustively searching all possible combinations of variables with the goal of discovering a well-fitting model that explains variance in an outcome of interest.

### Grammatical Evolution Neural Networks (GENN) and Domain Knowledge: ATHENA

Neural networks (NNs) are a robust and flexible modelling technique that attempt to mimic the basic structure and function of biological neurons to solve complex problems. NNs have been applied to many research fields, including robotics, speech recognition, optical character recognition, task scheduling, and industrial processing among many others. NNs have also been widely applied to various problems in biological science, including microarray data analysis [25], genotype calling [26,27], human linkage analysis [28], genetic association studies [29], medical expert systems [30], survival analysis [31], and protein folding [32]. The conventional approach for applying NNs to a classification problem is to specify a network architecture, select which variables (SNPs) are included as inputs to the network, and fit network weights using a gradient-descent based approach such as backpropagation (BP) [33]. While BP is capable of quickly fine-tuning weights in a NN, variable selection and modelling are goals which cannot be accomplished using this traditional approach. Recently, numerous evolutionary search strategies have been applied to NN classification problems to reduce the issues associated with the traditional NN approach [34]. Genetic Programming Neural Networks [35] and Grammatical Evolution Neural Networks (GENN) [36] use genetic programming [37] or grammatical evolution (GE) [38] to evolve populations of neural networks for human genetics classification problems. These populations are a heterogeneous mix of architectures, weights, and input variables which undergo mating, crossover, and recombination to ultimately identify an optimum NN solution, simultaneously finding influential SNPs and fitting networks weights. Recent work has shown that certain features characteristic of human genetic data may provide advantages to methods that evolve NNs to detect gene-gene interactions by transforming the fitness landscape from a "needle in a haystack" to a broader, smoother surface [39].

The application of GE to find epistatic gene-gene interactions is still exceedingly difficult, especially when the underlying disease model is purely epistatic, where each variant has no independent effect on the phenotype [40]. After demonstrating the critical need for expert knowledge when applying genetic programming to GWAS [41], others have shown that using expert knowledge guided mutation, selection, and crossover is highly beneficial, and dramatically improves the performance of evolutionary algorithms [42,43]. In much of the previous work showing that expert knowledge increases the performance of natural computing algorithms for finding epistatically interacting SNPs, the statistical expert knowledge was gleaned intrinsically - typically using a data-driven approach using variants of the Relief algorithm for feature selection [43-45].

Here we extend our previous work with NN training [46] to evaluate several fundamental modifications to the algorithm in a new tool, ATHENA (the Analysis Tool for Heritable and Environmental Network Associations). First, we implemented an alternative tree-based GE crossover strategy as previously described [46,47]. A potential weakness of GE is the destructive single-point binary crossover (SPBXO) operator [38]. Tree-based crossover (TBXO) instead swaps functionally analogous branches by first translating the grammar into functional neural network trees, identifying branches with identical root nodes, then initializing a crossover back at the genome level which would correspond to the crossover between the whole branches. This renders GE to be much more like genetic programming (GP), while still maintaining some of the key advantages of GE. We also evaluate the performance improvement when we combine GE with the traditional approach of fitting network weights with backpropagation. Finally, we evaluate with simulation whether utilizing available biological domain knowledge gleaned extrinsically would increase ATHENA's performance in discovering epistatic interactions between genetic variants contributing to a quantitative trait outcome. Here we present results of a simulation study showing that (1) using an alternative crossover strategy (TBXO) results in a considerable performance increase in some scenarios, (2) a hybrid backpropagation-GENN training algorithm has better performance than GE alone, and (3) incorporating biological knowledge from external sources results in an increase in ATHENA's ability to detect and model gene-gene interactions among a large pool of unassociated noise variables.

## Methods

### Genetic data simulation with genomeSIMLA

Simulated data where the true identity and size of the genetic or environmental effect in the population is known is a necessity for developing and testing novel methodology. It is also important that these true effects are embedded in a dataset containing many other nonfunctional polymorphisms and environmental factors, as is the case when real genetic data is collected. We developed genomeSIMLA [48] for simulating genome-wide scale data in population based case-control samples with a categorical outcome. Here we use an extension of genomeSIMLA capable of simulating gene-gene interactions in the presence of main effects, all of which influence a quantitative trait at a desired effect size [46]. The genomeSIMLA source code and binaries can be downloaded freely online [49].

Whereas the common measure of effect size in genetic association studies employing a case-control design is the odds ratio, studies of continuously distributed outcomes, such as HDL cholesterol level, estimate effect size as the proportion of variance explained [8,50], or R^2^. This variance explained, or heritability, can be further divided into genetic and nongenetic components, and the genetic component can be further divided into additive, dominant, and epistatic variance components [51]. The variance component explained uniquely by a single source of genetic variation (e.g. the main effect of one member of an interacting pair of variants, or the epistatic effect of the interaction term) is given by the semi-partial squared correlation coefficient [52]:

$$sr_{i}^{2}=R_{Y.x_{1},x_{2},...x_{i},...x_{k}}^{2}-R_{Y.x_{1},x_{2},...(x_{i}),...x_{k}}^{2}$$

The first term on the right side of the equation is the overall variance explained by fitting a full model (regressing the outcome, *y*, on each main effect and the interaction term between them). The second term on the right-hand side is the proportion of variance explained by the model when a predictor variable of interest is omitted from the model - for instance, omitting the interaction term. The difference between these two quantities is the semi-partial squared correlation coefficient [52], and describes the unique impact on the phenotype, *y*, for the particular variance component, *x_i_*. These estimates do not take into account the bias corrections discussed by Boerwinkle and Sing [50]. As these investigators showed, the bias in these estimators for the number of genotype classes represented here quickly approaches zero as sample size increases past n = 100. Since our simulated datasets comprise 2000 samples, the bias discussed by these investigators is essentially zero.

Datasets were simulated as previously described [46]. Briefly, samples are drawn from a homoscedastic normal distribution with the mean being determined by the genotypes at the corresponding functional genetic variants. We simulated 500 SNPs in 2000 samples, where only two SNPs were functional and the other 498 SNPs were unassociated "noise" variables. We simulated a gene-gene interaction between these two SNPs that carried a narrow-sense heritability (h^2^) of 0.05, meaning that only 5% of the variation in the quantitative trait could be explained by this gene-gene interaction. This low effect size is typical of most findings in human genetic epidemiology [18,19]. We simulated this interaction in the context of very small main effects at each locus (h^2 ^= 0.01). Both main effects and the gene-gene interaction were additive. A scenario such as this where main effects explain little of the overall outcome variance represents a very difficult problem [53] for an evolutionary search procedure to model.

### Domain knowledge

A recently developed tool called Biofilter is capable of integrating information from several publicly available biological databases in order to assess specific combinations of genetic variations and their effect on the outcome based on prior statistical and biological knowledge [54]. Specifically, this tool uses the Gene Ontology [55], the Database of Interacting Proteins [56], the Protein Families Database [57,58], the Kyoto Encyclopedia of Genes and Genomes (KEGG) [59], Reactome [60], NetPath [61], and the Genetic Association Database (GAD) [62] in order to construct two-SNP models that are supported by the biological literature. Their degree of support in the literature is characterized by an implication index - which is a count of how many times a relationship between a pair of two genes appears across multiple databases incorporated into Biofilter.

To determine whether incorporation of domain knowledge into NN training in ATHENA can improve its performance, simulated domain knowledge that mimics information obtained from Biofilter must be generated. Here, 4000 random undirected edges are drawn between a subset of the 500 SNPs simulated as described above. The implication index is the number of edges drawn between two models. This number typically ranges from 0 to 5, where implication index of zero indicates no support in the simulated knowledge pool, while an implication score of 5 indicates that this model is very well supported. The implication index corresponding to the functional two-SNP model where the true effect was embedded could be manually specified. Our specific goals were to determine if and to what degree ATHENA's performance would diminish if irrelevant domain knowledge were incorporated, and if and to what degree ATHENA's performance would increase if accurate domain knowledge were incorporated into the training process.

### Alternative crossover and incorporation of domain knowledge in ATHENA

**Figure 1:**
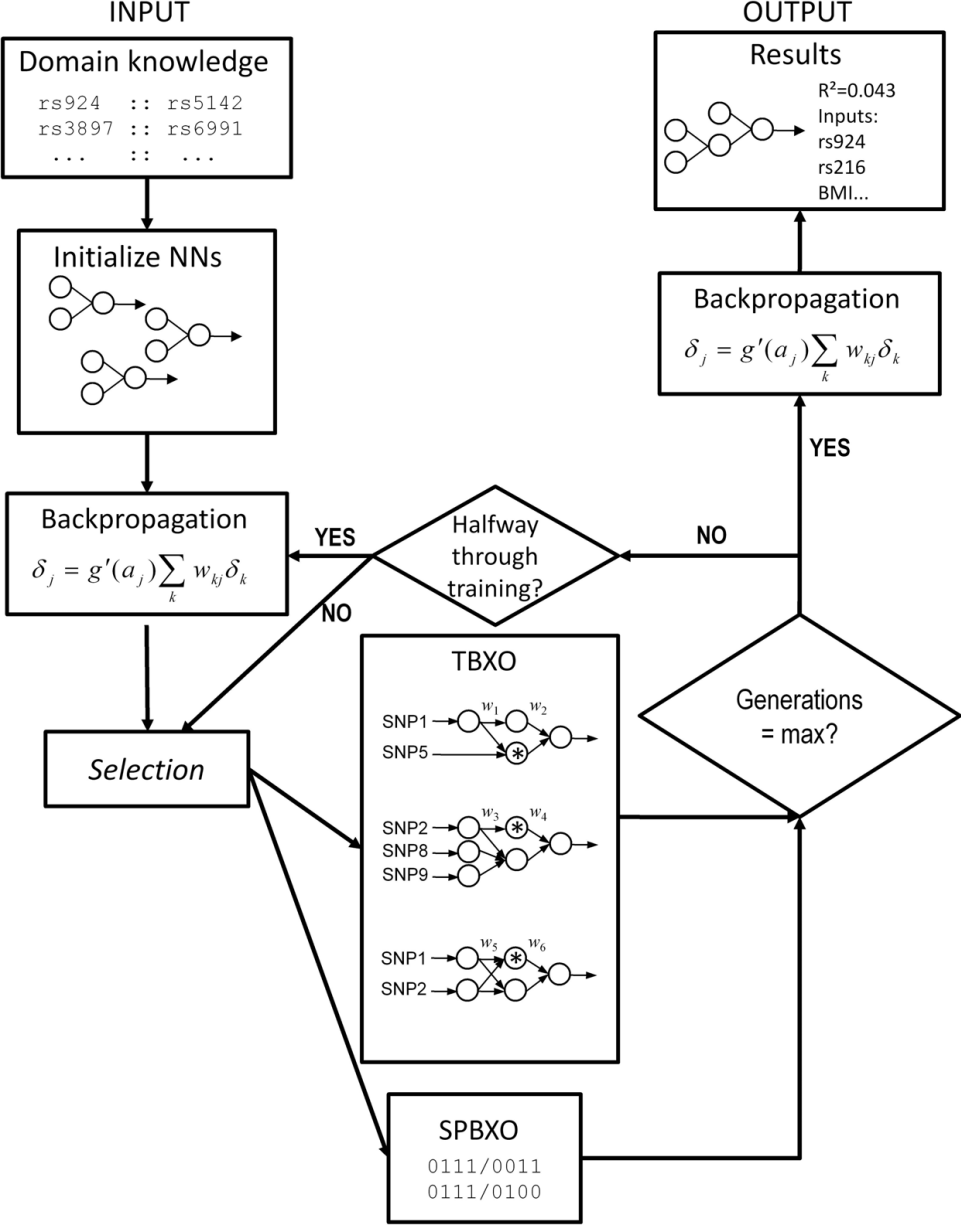
**ATHENA algorithm**. The ATHENA algorithm begins by optionally accepting a list of SNP-SNP models which are derived from biological knowledge sources. This domain knowledge is used to initialize a proportion of the NN population. BP is used to optimize the initial weights. After a round of selection, GE is used to simultaneously optimize variable selection, NN architecture, and weights. Another round of BP takes place midway through training, and at the end of training. Crossover can occur via single point binary (SPBXO) or tree-based crossover (TBXO). In SPBXO, crossover occurs at the binary string level, but in TBXO, NNs are first translated, and crossover occurs at the binary genome level that results in a crossover at functionally similar root nodes. The NNs in this figure have either two or three inputs, corresponding to numerically coded values (-1, 0, 1) for SNP genotypes. A weight vector corresponds to each layer of weights in the NN. In TBXO, functionally analogous branches are crossed over, indicated by the asterisk, resulting in a 2-2-1 neural network with SNPs 1 and 2 as inputs. If SNPs 1 and 2 are the functional SNPs responsible for the gene-gene interaction and if the weight vectors on this NN are favorable, then this NN should be capable of modelling a gene-gene interaction between these two SNPs.

NN training in ATHENA has been implemented as previously described [36,46]. Briefly, grammatical evolution (GE) is a variation of genetic programming (GP), an evolutionary algorithm originally proposed by Koza as a procedure to optimize NN architecture [37]. In GE, randomly initialized binary strings are transcribed into an ordered list of integers which are used to select from production rules in a Backus-Naur form grammar. Our grammar applies GE to construct neural networks, and can simultaneously select important predictor variables and optimize network weights and architecture. We also implemented an alternative tree-based GE crossover strategy as previously described [46,47]. A potential weakness of GE is the destructive single-point binary crossover (SPBXO) operator [38]. Tree-based crossover (TBXO) instead swaps functionally analogous branches by first translating the grammar into functional neural network trees, identifying branches with identical root nodes, then initializing a crossover back at the genome level which would correspond to the crossover between the whole branches. This renders GE to be much more like genetic programming (GP), while still maintaining some of the key advantages of GE. Representative NNs produced by GE, and the TBXO process are shown in Figure 1, under the "TBXO" panel. The NNs in this figure have either two or three inputs, corresponding to numerically coded values (-1, 0, 1) for SNP genotypes [63]. A weight vector corresponds to each layer of weights in the NN. In TBXO, functionally analogous branches are crossed over, indicated by the asterisk, resulting in a 2-2-1 neural network [64] with SNPs 1 and 2 as inputs. If SNPs 1 and 2 are the functional SNPs responsible for the gene-gene interaction and if the weight vectors on this NN are favorable, then this NN should be capable of modelling a gene-gene interaction between these two SNPs.

In the first set of experiments, ATHENA was run for 100, 200, and 400 generations, in runs consisting of population sizes 100, 200 and 400, in each of 10 demes (for a total NN population size of 1000, 2000, and 4000 respectively), on 100 simulated datasets. We varied the number of generations where tree-based crossover was used (TBXO). This could range from using single-point binary crossover (SPBXO) for every generation (i.e. no TBXO), TBXO for the first half of the total number of generations before switching back to SPBXO, or TBXO for the total number of generations run. This resulted in trials using 54 different combinations of ATHENA parameters, comprising 5,400 *in silico *datasets. The mean runtime per dataset was approximately 14 minutes spread across five 1.8 GHz Opteron PCs. The respective probability of a crossover and mutation were 0.9 and 0.01, typical values for these parameters in many genetic algorithms [65]. Addition was the only production rule available for the arithmetic operator at each activation node, as described previously [46,63]. This allowed for the implementation and optional usage of backpropagation (BP), a local fitting procedure designed to optimize the weights in a neural network [33]. BP was either not used at all, or used at initialization and again at generations 100 and 200, using a learning rate of 0.3. BP was halted after either a maximum of 100 epochs had been run, or when further BP showed no improvement (mean squared error is reduced by less than 1 × 10^-6^), after which the GE process continues. After every network had undergone BP, NNs were reverted back to a binary genome by marking blocks of codons corresponding to a weight, which was then replaced with a block containing a grammar compatible block that generates the appropriate weight when GE continues after BP.

In the second set of experiments, domain knowledge was used to perform sensible initialization. Rather than initializing a population of NNs randomly, the initial generation is partially composed of NNs containing as input variables SNPs that are represented in a domain knowledge source. This source can be two-SNP models supported by biological literature derived from Biofilter [54] or simulated domain knowledge which mimics domain knowledge derived from Biofilter. Part of the population is still initialized randomly. Here the proportion of the initial population which is initialized from domain knowledge was varied from 0 to 99% in intervals between 1-10%. Two-SNP models from domain knowledge are prioritized for incorporation in the initial generation based upon implication index - models with higher implication index are initialized first. The implication index on the functional two-SNP model in these experiments ranged from 0 (negative control - all domain knowledge incorrect/irrelevant) to 3 (functional two-SNP model is somewhere in the top half of the implication index-ranked list of 4000 domain knowledge two-SNP models). Here, ATHENA was run for 200 generations using 10 demes with population sizes of either 50, 100, or 200 individual NNs. The mean runtime per dataset was approximately 6 minutes spread across five 1.8 GHz Opteron PCs. As above, probability of a crossover and mutation were 0.9 and 0.01, and the production rule for the activation node function was restricted to addition only, which allowed for the optional use of backpropagation. In addition to locally fitting weights to improve the model fit when NNs are initialized randomly, this hybrid algorithm allows for weight optimization in the event that sensible initialization from domain knowledge resulted in the inclusion of either of the two functional variables in the initial generation.

## Results & Discussion

For the *in silico *studies described above, sensitivity was measured as the proportion of datasets out of 100 simulated datasets for each scenario where the best performing neural network model contained the two functional SNPs, with no other SNPs in the model, i.e. a perfect match [46]. The best neural network model for each dataset was chosen using the following algorithm. First, 5-fold cross-validation (CV) was implemented. The data is divided into fifths, training initially occurs on four fifths of the data where a best model is chosen based on minimizing mean square error. The fit of this model to unseen data was tested on the fifth of the data initially left out using the standard coefficient of determination, R^2^. This process was repeated for each CV interval, i.e., each 4/5-1/5 split of the data. At this point there are 5 models - one best model from each CV interval. The model that consistently appears most often across CV intervals is chosen as the best overall model for the entire dataset [16,66]. In case of a tie (e.g. two different models replicated across two CV intervals), the model with the higher R^2 ^is chosen as the overall best model.

### Tree based crossover (TBXO)

**Figure 2:**
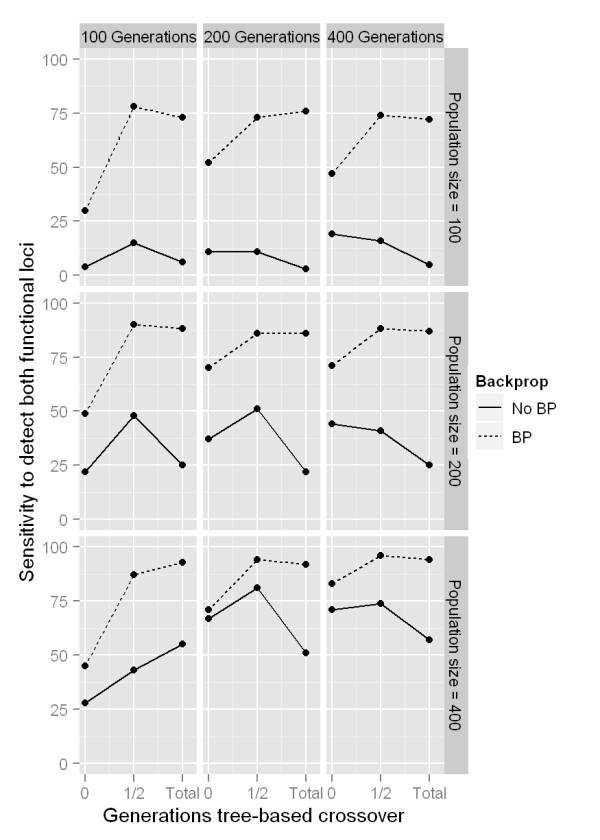
**Sensitivity to detect both functional loci as the best GENN model**. Each panel shows sensitivity over the proportion of total generations (none, half, all) where tree-based crossover was used instead of binary crossover. Solid line shows when GE alone was used to train NNs (no BP). Dashed line shows sensitivity when using the hybrid BP-GENN algorithm (see methods). Individual panels show combinations of the total number of generations GENN was run and the population size per deme.

**Figure 3:**
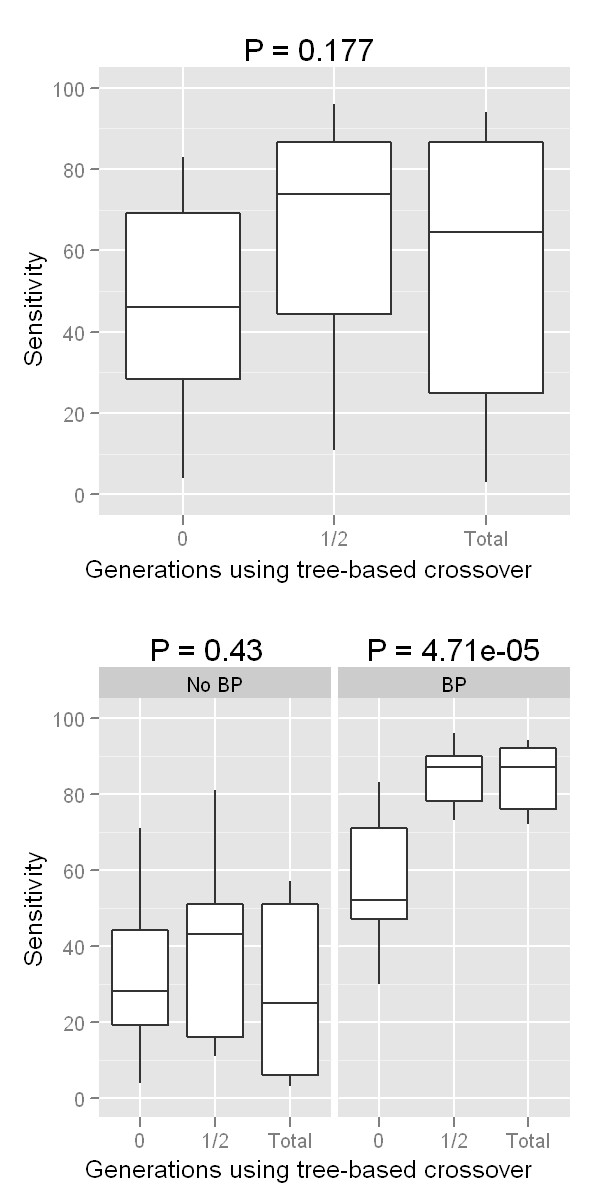
**Statistical analysis of effectiveness of tree-based crossover**. Boxplots show the distribution of sensitivity across all combinations of generations run and population sizes (see Figure 2). P-values indicate whether there is a statistically significant increase in sensitivity gained by using TBXO (one-way analysis of variance). Top panel shows the combined results from using and not using backpropagation. Bottom panel shows the results considering simulations using and not using backpropagation independently.

First we wanted to evaluate whether the alternative TBXO strategy described in the methods section resulted in increased performance in the context of GE alone or with the hybrid BP-GE algorithm in ATHENA which also used backpropagation (BP) in addition to GE. These results are summarized in Figure 2. Separate panels show the total number of generations and the size of the population in each deme. Dashed and solid lines show the performance (sensitivity) when BP was and was not used, respectively. The horizontal axis on each panel shows the proportion of the total number of generations in which TBXO was used. These results also show that our implementation of TBXO yields a modest yet notable increase in sensitivity, but when BP was not used, the performance increase is observed only when TBXO is used exclusively in the early generations of training (see the center point in the solid lines in each panel in Figure 2). When BP was used in addition to GE to locally fit NN weights, using TBXO for the first half of training resulted in increased performance that did not change when TBXO was used throughout the rest of training (dashed lines in Figure 2). This is in contrast to previous work where TBXO showed little improvement when the simulated model was an interaction contributing to a discrete trait in the complete absence of main effects [47]. We then statistically evaluated this performance increase, summarized in Figure 3. Here, boxplots show the distribution of sensitivity across all combinations of generations and population sizes, and P-values indicate whether there is a statistically significant increase in sensitivity gained by using TBXO (one-way analysis of variance). The top panel of Figure 3 shows the combined results from using and not using backpropagation. Bottom panel shows the results considering simulations using and not using backpropagation independently. This indicates that the benefit from using TBXO when concurrently using BP is highly statistically significant, but there is little evidence to suggest using TBXO with the standard GE crossover alone results in any appreciable performance gains.

These results indicate that when BP is used, GE with TBXO is more efficient at variable selection, while GE with normal crossover allows more variation in building architecture and fitting weights. We postulate that TBXO is preserving "building blocks" which are functionally useful to the resultant neural network models. Our simulations contained a modest interaction effect (h^2 ^= 0.05) in the presence of very small main effects (h^2 ^= 0.01) at each of the interacting genetic variants. These small main effects may provide the building blocks upon which TBXO can capitalize. Syntactic preservation of NN genomes coding for the inclusion of these variables in NN models while allowing the full variability and broader search capability of SPBXO in the latter generations of evolution appears to be more powerful than using SPBXO or TBXO exclusively. Furthermore, recent work has shown that linkage disequilibrium (correlation between genetic variants) may provide building blocks to an evolutionary algorithm which builds neural networks when the true underlying model is an interactive effect in the complete absence of any main effect at each of the two functional variables [39]. It is expected that the TBXO strategy discussed here may be optimal in this situation as well. Because our TBXO procedure mimics the function of genetic programming (GP), further studies should compare this against GP or any hybrid GP-GE NN training algorithm.

### Incorporation of domain knowledge

**Figure 4:**
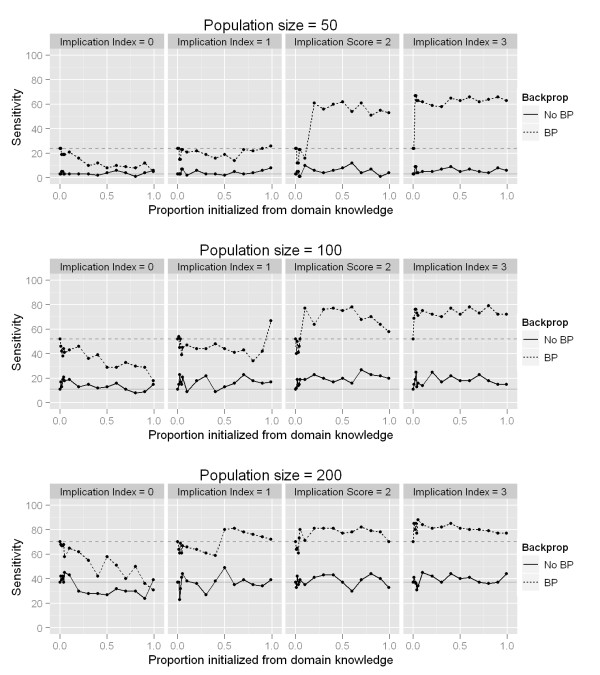
**Sensitivity increases with the proportion population initialized from domain knowledge**. This figure illustrates the sensitivity of GENN to detect both functional SNPs as the proportion of the NN population initialized from domain knowledge increases from 0 to 99%. Panels going left to right show the increasing implication index of the model that includes both functional variables. Rows of panels show the population size per deme. The X-axis in each panel shows the proportion of the initial NN population which was seeded with two-SNP models from a domain knowledge source. Solid line shows when GE alone was used to train NNs (no BP). Dashed line shows sensitivity when using the hybrid BP-GENN algorithm (see methods). Faint horizontal solid and dashed lines show for reference the baseline sensitivity, for GENN and BP-GENN, when the population was initialized randomly, i.e. 0% initialized from domain knowledge. This figure indicates that sensitivity increases as the proportion of the NN population initialized from domain knowledge increases, and the increase is more notable in smaller population sizes.

Next we evaluated whether initializing the NN population with two-SNP models from domain knowledge sources resulted in any changes in performance. These results are summarized in Figure 4. The results here show that sensitivity to detect both genetic variants contributing to the trait is always higher when BP was used in conjunction with GE, as also shown in Figure 2. When the implication index is 0 (i.e. all domain knowledge is irrelevant), the sensitivity when using BP decreases substantially as the proportion of the initial population initialized from domain knowledge increases (upper left panel of Figure 4, dashed line). This is likely due to the fact that as more NN models are initialized from a list of models from irrelevant domain knowledge, there is a smaller chance that either of the functional variables can be initialized by chance. When the implication index is at least 1 (meaning the functional two-SNP model is supported in our domain knowledge), as this proportion increases, sensitivity fluctuates around the baseline sensitivity (37%) at random initialization when BP is not used. This is not surprising, because even if a NN is initialized containing both functional variables which influence the trait, it is unlikely that by chance the NN would have suitable weights and architecture. An increase in performance can be seen when BP is then used to optimize the weights in the sensibly initialized NNs from relevant domain knowledge (dashed lines in panels in Figure 4 where implication index > 0). Furthermore, as the implication index for the domain knowledge model containing the functional variables increases from 1 to 3, this model is more likely to be incorporated into NNs in the initial generation. For instance, when the implication index of the functional model is 1, approximately 99% of the population must be initialized from domain knowledge in order to see any benefit. When the implication index is 2 or higher, it is very likely that the initial generation will contain a NN with the truly functional variables even when only a small proportion of the initial population is initialized from domain knowledge. Finally, looking down the rows of panels in Figure 4, it is clear that although the overall performance increases as the population size increases, as expected, the benefit of utilizing domain knowledge becomes less apparent. The benefits gained from utilizing domain knowledge to initialize a population of solutions is most apparent when the search space is large relative to the number of candidate solutions, as seen in the top right panel (implication = 3, population size = 50), dashed line.

These results demonstrate that the sensitivity of using GE to train NNs to find genes with a nonlinear influence on a quantitative outcome can be improved by effectively using extrinsic domain knowledge in conjunction with local weight fitting by BP. We showed that initializing a proportion of the NN population from two-SNP models incorporated from domain knowledge when BP is employed to locally optimize the weights in a NN can result in a performance improvement in ATHENA's ability to detect and model SNPs influencing a quantitative trait. The performance increase was most notable when a smaller population size was used. This indicates that when the search space is small enough to be searched very thoroughly or exhaustively, using domain knowledge is less beneficial than when the search space is very large compared to the number of individual solutions being evolved. In this scenario (such is the case in genome-wide association studies), using domain knowledge to bias an evolutionary search in favor of important features will be critical for acceptable performance. While the benefits of using intrinsically obtained statistical expert knowledge [42,43] have not been explored in the ATHENA algorithm, using this framework to initialize an evolutionary search for disease genes based on domain knowledge obtained from public biological databases is another means to improve the performance of genetic algorithms for selection of important SNPs in a model.

### Comparison with other methods

As discussed in the background section, other methods are available for probing the effect of gene-gene interactions on quantitative phenotypes. One exhaustive approach to testing gene-gene interaction among quantitative traits is the restricted partitioning method (RPM)[67], an improvement over the combinatorial partitioning method [68]. RPM exhaustively evaluates all possible combinations of 2, 3,..., n-way combinations of SNPs, restricting the partitioning of each multilocus genotype into subsets that are likely to explain the most variation. While RPM should have high power and favorable computational performance in small datasets, as with any exhaustive approach to detecting interactions, its performance will decrease substantially as the number of SNPs in a dataset approaches that seen in genome-wide association studies. In addition to being extremely computationally intensive, exhaustive evaluation of all possible SNP-SNP interactions among GWAS data comes with an extraordinary loss of power due to the extremely large number of statistical tests being performed. Alternatively, parametric linear regression, when assumptions of normality and homoscedasticity are met, is uniformly the most powerful statistical method for ascertaining differences in group means [52,69]. In fact, when the functional variables are explicitly modelled, linear regression has >80% power to detect the gene-gene interaction effects simulated here (*n *= 2000, *sr^2^_main _*= 0.01, *sr^2^_interaction _*= 0.05), determined using standard power calculation techniques for gene-gene interaction [70,71]. This regression-based interaction-testing approach has been successfully used in a study of 13 SNPs in the APOE gene that influence ApoE protein levels in the blood [72]. Furthermore, regression models offer a very straightforward interpretation compared to NN models, which are often and unfortunately dubbed "black box" models [73]. However, in addition to the disadvantages discussed previously (curse of dimensionality, assumption violations, computational and multiple testing burdens with large datasets) that make exhaustive regression-based approaches impractical, it is also difficult to incorporate *a priori *information into a parametric regression analysis as it has been done here. Several approaches have been applied to prioritize gene-gene interaction testing [54,74-76] in large datasets. These methods, however, limit statistical tests only to models supported by *a priori *knowledge. By contrast, the method proposed here only initializes a set of candidate solutions using domain knowledge - these solutions are then free to mutate and crossover, resulting in new and interesting combinations that may not be directly supported by the existing domain knowledge.

## Conclusions

Here, we simulated a small effect size nonlinear interaction between two SNPs carrying minimal main effects and assessed the sensitivity of using GE to evolve NNs for detecting both functional SNPs out of a much larger set of unassociated variables. We showed that (1) using backpropagation, a fast NN weight optimization procedure, significantly improves ATHENA's performance, (2) using an alternative crossover strategy (TBXO) may allow for functional preservation of network information, and results in a statistically significant performance increase when used early in training in combination with backpropagation, and (3) incorporation of biological knowledge from the public domain can substantially improve ATHENA's performance at finding genes that interact to influence a trait. The general ATHENA algorithm is shown schematically in Figure 1.

Supplementing an evolutionary search using domain knowledge will be critical when using evolutionary procedures to find and model the effect of disease genes on complex human traits. Natural biological data will likely have many effects which will be enriched in knowledge sources, resulting in an improvement of the overall ability to find many members in the collection of influential loci. Genome-wide association studies offer very inexpensive measurement of over 1 million SNPs per sample. It is clear that there are more fruitful approaches for understanding the genetic architecture of common human phenotypes than ignoring the complexity of biology by testing single variants in isolation [77]. One of the strengths of the method presented here is that if any arbitrarily complex interaction of genetic and environmental exposures influences disease risk, a NN can approximate this function [78], given proper training. These experiments show that using a hybrid BP-GENN training algorithm, alternative crossover strategies, and incorporating domain knowledge into the search for genes related to disease can aid the variable selection and model fitting process of ATHENA.

One limitation in the current study is that these experiments make the assumption that loci involved in gene-gene interactions contributing to a heritable trait will carry with them some small main effect at either variant. This is a reasonable assumption to make, in that there are few, if any, examples of a consistently replicating, experimentally verified gene-gene interaction in the complete absence of main effects contributing to a complex quantitative trait in humans. Perhaps the reason for this, however, is the inadequacy of our methods for finding gene-gene interactions in the absence of main effects rather than the absence of such effects altogether. Biologically, redundancy and compensatory mechanisms at other loci can mitigate the effects of a devastating mutation or polymorphism at another locus, thus rendering its effect undetectable. This is evident in the many gene knockout mouse lines that show no apparent phenotype [79-84]. Statistically, main effect components and interactions between them are mathematically independent effects [69]. Furthermore, theoretical studies have shown that traits can be influenced exclusively through the interaction of two or more genetic variants [85,86]. Finally, one group has shown that main effects at variants involved in an epistatic interaction are highly dependent on the allele frequency in different populations at each locus, which may explain the lack of replication of many gene-gene interaction studies which rely on main effects [87]. Future studies should aim to assess these and other extensions of ATHENA in their ability to detect and model epistatic interactions contributing to a quantitative trait in the absence of main effects, and should attempt to apply these methods in a natural biological data analysis.

## Competing interests

The authors declare that they have no competing interests.

## Authors' contributions

SDT performed the simulations and sensitivity analyses presented in this work. SMD designed and implemented the software presented here. SDT & MDR conceived of the study, and participated in its design and coordination and drafted the manuscript. All authors read and approved the final manuscript
